# Robust Ship Detection in Infrared Images through Multiscale Feature Extraction and Lightweight CNN

**DOI:** 10.3390/s22031226

**Published:** 2022-02-06

**Authors:** Rui Miao, Hongxu Jiang, Fangzheng Tian

**Affiliations:** Beijing Key Laboratory of Digital Media, School of Computer Science and Engineering, Beihang University, Beijing 100191, China; miaor@buaa.edu.cn (R.M.); amazingtian@buaa.edu.cn (F.T.)

**Keywords:** ship detection, multiscale feature extraction, lightweight CNN, robustness

## Abstract

The sophistication of ship detection technology in remote sensing images is insufficient, the detection results differ substantially from the practical requirements, mainly reflected in the inadequate support for the differentiated application of multi-scene, multi-resolution and multi-type target ships. To overcome these challenges, a ship detection method based on multiscale feature extraction and lightweight CNN is proposed. Firstly, the candidate-region extraction method, based on a multiscale model, can cover the potential targets under different backgrounds accurately. Secondly, the multiple feature fusion method is employed to achieve ship classification, in which, Fourier global spectrum features are applied to discriminate between targets and simple interference, and the targets in complex interference scenarios are further distinguished by using lightweight CNN. Thirdly, the cascade classifier training algorithm and an improved non-maximum suppression method are used to minimise the classification error rate and maximise generalisation, which can achieve final-target confirmation. Experimental results validate our method, showing that it significantly outperforms the available alternatives, reducing the model size by up to 2.17 times while improving detection performance be improved by up to 5.5% in multi-interference scenarios. Furthermore, the robustness ability was verified by three indicators, among which the F-measure score and true–false-positive rate can increase by up to 5.8% and 4.7% respectively, while the mean error rate can decrease by up to 38.2%.

## 1. Introduction

Ship detection from infrared remote sensing images has an important but challenging role in remote surveillance and military reconnaissance [1,2]. Due to the large coverage area of infrared remote sensing images, and the small proportion of targets in the images, the accuracy of target-detection algorithms and real-time processing performances is seriously affected. For example, a remote sensing image obtained by one satellite contains 30,000 × 30,000 pixels, among which the size of ship area is 10 × 10 pixels, and the target area only accounts for one part per million of the image, which results in a serious lack of target texture detail, especially when the image resolution is low. Although certain existing infrared-image ship detection technologies have emerged, it is difficult for these methods to simultaneously address the following challenges: (1) it is difficult to extract universal features for various types of target ships in low-resolution or low-contrast images. Detection in low-resolution or low-contrast images will lead to the loss of texture details, and difficulty in the extraction of the available features of the target. Coupled with the interference of infrared image noise from clouds, reefs and so on, the features of the middle and lower layers are easily ignored, and it is easy to generate false-positives and negatives. (2) It is harder to maintain the balance between algorithm performance and algorithm complexity. Generally speaking, algorithms with high detection performances have high levels of complexity due to their calculation modes. When algorithms with high complexities need to be transplanted to embedded chips with limited resources, part of the algorithm’s performance is often sacrificed. (3) Most of the existing detection methods compromise on real-time computing. The majority of detection algorithms have requirements that make them difficult to match with the resources that are available in real-time space reconnaissance applications, especially when introducing algorithms with the depth of neural networks to target classification; it not only requires a large amount of training data to be effectively generalized but also uses a larger amount of computational power compared to other methods.

Infrared images have a strong spatial correlation, contain more homogeneous regions, and have weak texture features, so the mean gray value is relatively stable [3]. However, when detecting a target ship on the sea surface, there are still several forms of interference: first of all, because the response characteristics of each pixel in the infrared imaging equipment are not completely consistent, different detection units produce different outputs under the same radiation input, so there is a bright and dark striped noise in the infrared image, resulting in a low signal-to-noise ratio of the image, which seriously affects the performance of the target-detection algorithm. Secondly, remote sensing satellites are easily affected by clouds, sea-clutter, and other weather during imaging, resulting in a complex detection background, reduced contrast between the target and background, which makes it easy to produce a large number of false-alarm. Thirdly, it is difficult to select appropriate features to separate the target and background because of the existence of various types and sizes of targets, and the unequal representation of grayscale features. Therefore, at present, there is no detection algorithm that is applicable to all possible scenarios, exist methods can only be used to minimise interference and ensuring detection efficiency in specific detection scenarios.

With the improvement of artificial intelligence technology, deep learning can adaptively and automatically learn features in data by constructing a deep neural network, which makes up for the deficiency of manual design features to a certain extent [4,5,6]. Object detection technology based on deep learning can generally be divided into one-stage and two-stage detection methods. Two-stage target-detection algorithms generally consist of candidate-region extraction and target confirmation, they utilize powerful features with statistical classifiers to discriminate ships from false-alarm, which has great advantages in maintaining detection accuracy. One-stage target-detection algorithms aim to extract all the candidate regions for a subsequent classifier, they omit the step of candidate-region extraction and directly obtain the target category and position from the image. Compared with two-stage algorithms, they have a huge speed advantage, but their detection accuracies are low due to their rough detection strategy. When deep learning algorithms are deployed on embedded platforms or other platforms, it is a great challenge to balance the accuracy, speed and memory resources needed for target detection.

As a result of the above-mentioned analysis, the complex background and diverse interference factors will seriously affect the extraction and classification of the effective features of the targets. In addition, during the process of deployment and application, it is difficult to balance detection performance, computational complexity and real-time performance. In order to solve the problems raised above, the contributions of this paper are presented as follows. (1) Candidate-region extraction: our method combines the cascade rejection mechanism with multiple other features through a linear cascade classifier, which orders candidates from simple to complex, uses relatively simple features to exclude a large number of simple alarms, such as seawater and clouds, and uses more sophisticated features to extract final candidate regions. (2) Multiple feature fusion-classification: a false-alarm elimination method based on Fourier global spectral features and lightweight CNN is proposed. In this method, the global Fourier transform was applied to each candidate area to obtain the corresponding feature description and achieve a rough classification of candidate regions, then the local feature was extracted by the lightweight CNN model to further eliminate false-alarm. (3) Classifier training and target confirmation: a classifier training algorithm is proposed to minimise the classification error rate and maximise generalisation, and the improved NMS algorithm is used to merge real ships and achieve an accurate output.

The remainder of this paper is organized as follows. Section 2 describes the related work. Section 3 introduces the methodology and details the elaborate implementation and optimization of the core module of the algorithm. Section 4 describes the extensive experiments, and Section 5 presents our conclusions and recommendations future work.

## 2. Related Work

In recent years, many infrared ship detection algorithms have been proposed by researchers. In these researches, ship target detection algorithms are generally divided into traditional ship detection method and deep learning ship detection method. The existing traditional ship detection algorithms can be divided into four categories for different scenarios: ship detection algorithms based on wake extraction; ship detection algorithms based on template matching; ship detection algorithm based on feature statistics; and ship detection methods based on classification learning. Although some scholars have put forward some novel research ideas, their core ideas are inseparable from the above types of target detection. Traditional target-feature extraction methods are mainly based on the idea of grey and texture features where a pre-trained classifier is employed for classification. For example, some researchers propose a saliency strategy, a feature descriptor [7,8], and a local comparison method [9] to determine small infrared targets, but these methods are very sensitive to noise, which usually generates a high false-alarm rate. Most infrared small-target-detection methods based on saliency have high computational complexity and are difficult to optimise using parallelism. Therefore, ship detection algorithms based on the weighted local difference measurement [10], and weighted voting mechanism [11] have been proposed. Moreover, references [12,13] introduce the multiscale local uniformity and greyscale difference weighting strategy to detect small infrared targets. In references [14,15], multi-frame images and sensor data in the infrared image sequence were analysed for ship detection. Based on extreme value theory, the edge detection [16,17] and cascading characteristics methods [18] are adopted to identify the objects of interest and suppress background clutter. The above traditional detection methods are often based on low-level, hand-made features. It is a great challenge to achieve high detection accuracy in complex scenes, such as those with cloud interference and low contrast, and improvement is needed.

With the improvement in artificial intelligence technology, deep learning has attracted an increasing amount of attention. Target-detection techniques based on deep learning include anchor-based and anchor-free techniques. Firstly, anchor-based technology includes one-stage and two-stage detection. One-stage detection techniques include the single-shot detector (SSD) [19], the deconvolutional SSD (DSSD) [20], RetinaNet [21], RefineDet [22], You Only Look Once Version 3 (YOLOV3) [23], etc. Two-stage detection techniques include FTP-region-based convolutional neural networks (RCNNs) [24], region-based fully convolutional networks (R-FCNs) [25], the Feature Pyramid Network (FPN) [26], Cascade R-CNNs [27], the subnet Internet Protocol (SNIP) [28], etc. Generally, two-stage target detection is more accurate than one-stage target detection, but the processing speed is slow. Secondly, anchor-free technology includes key-points and segmentation. Key-point-based technologies include CornerNet [29], CenterNet [30], Cornernet-Lite [31], etc., and segmentation technologies include a feature-selective anchor-free (FSAF) module [32], a fully convolutional one-stage (FCOS) object detector [33], FoveaBox [34], etc. These deep learning methods achieve good detection accuracy in natural image target detection, but they also have great limitations during satellite processing with limited remote sensing image resources. First, the compression method of deep neural networks has higher performance requirements, especially for large networks. Second, the algorithm has difficulty meeting performance expectations. It is difficult to design a state-of-the-art machine for the data-flow scheduling of different layers, and there will be considerable redundancy in logical resources. In addition, there is the data dependence problem. Compared with traditional methods, deep learning relies more on the large-scale training of data and it needs a large amount of data to understand the potential data-mode. When the target features and false-alarm features in the detected images are relatively uniform, such a data dependence problem is not obvious, otherwise, when the target features and false-alarm features in the detected images are significantly different, the scale of training data will need to be considerably increased, which is a great practical challenge.

In some relatively simple conditions, the methods mentioned above can achieve considerable detection results. However, the detection performance of these algorithms will be affected in the following three situations: (a) low contrast between ships and background; (b) scenes with complicated sea conditions; and (c) in situations of false-alarm interference. In addition, these algorithms also give rise to different levels of missed detection when multiple vessels are docked. Therefore, there is still much room for improvement in ship detection.

## 3. Methodology

### 3.1. Candidate-Region Extraction

**Figure 1:**
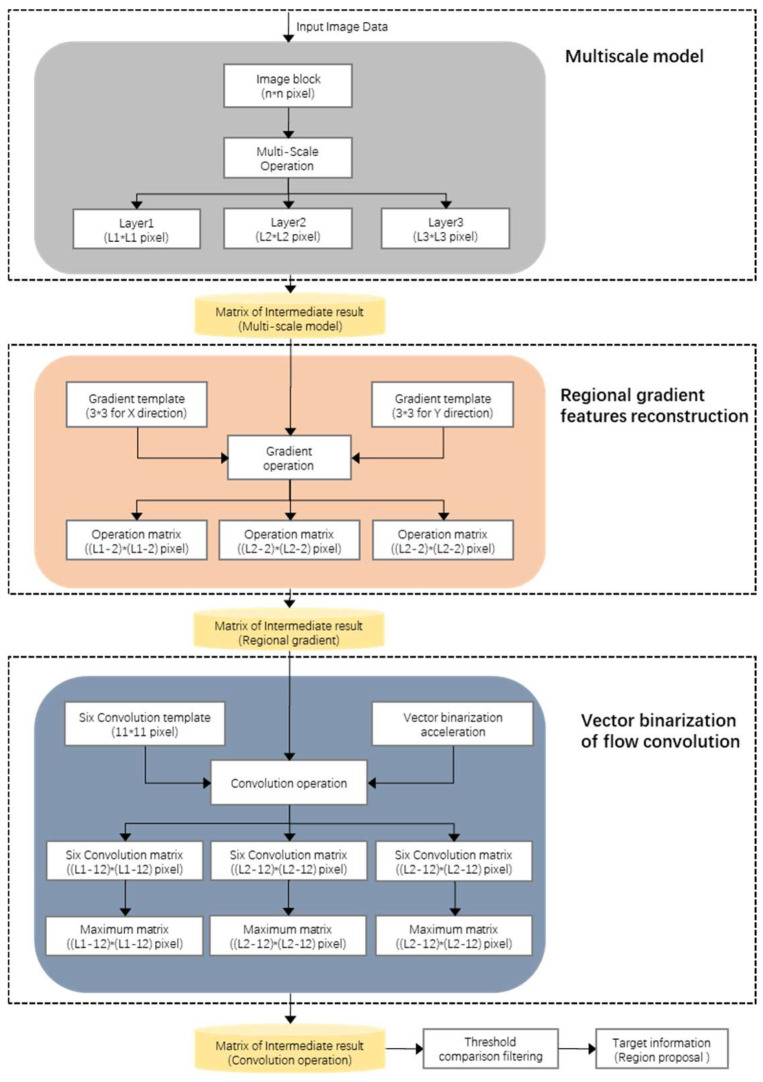
Schematic of the candidate-region extraction process.

To solve the problem of complete extraction of the ship region, the suspected region of the target ship is located step-by-step based on the idea of coarse-to-fine detection, as is shown in Figure 1. We design the candidate-region extraction through three parts. Firstly, using a multi-scale model, hierarchical images are constructed, which are employed as original image-data for subsequent processing. Secondly, regional gradient-feature reconstruction is undertaken. The region of interest (ROI) is extracted by constructing the Sobel operator and gradient template, and the target region is preliminarily determined. Thirdly, in the vector binarization of flow convolution, target information is extracted from the combination of multiple features by traversing the image data using flow convolution alongside using the more sophisticated features for further identification.

#### 3.1.1. Multiscale Model

The target types to be detected are different under different image resolutions, such as in 5-m-resolution images, the target ships are generally large military targets, large passenger targets and large cargo targets, but in high-resolution images, fishing ships and other small ships are also the targets that need to be detected. Ships in the same remote sensing image have different sizes, and the same ship has different scales in images with different resolutions. Therefore, the existing methods are difficult to adapt to problems that involve large differences in the apparent features of ship caused by scale diversity. In order to find target ships of different sizes in remote sensing images, we constructed a standard image model through repeated smoothing and sub-sampling, and then generated a multi-scale image model through reasonable scaling. The definition of each layer is shown in Equation (1).

(1) $${\mathrm{Lay}}_{i}={\mathrm{Lay}}_{\mathrm{ori}}⊗\theta_{i},i=1,2,3...n$$

where ${\mathrm{Lay}}_{i}$ represents the generated image models at different scales, ${\mathrm{Lay}}_{\mathrm{ori}}$ represents the standard image model, ⊗ represents the bilinear interpolation operation, $\theta_{i}$ represents the scaling factor, and the value of parameter i ranges from 1 to n. In the candidate-region extraction stage, one of the more important parameters is the filter size (k × k), which is the sliding cell that we need. As to the small target, we can set the filter size to ensure that it completely covers the target. As for the larger ships, if a smooth or partial calculation can’t cover the whole target, we down-sample the image according to the actual testing requirements in terms of narrowness, until the scaled target size meets the minimum coverage area. Therefore, the selection of the parameter “k” is very important. If k is too small, the target ship cannot be covered, otherwise, false-alarm will appear in the covering box. By analyzing the size of target ship in the dataset, we can select parameters 10–15, basically to detect all ships in the dataset on the basis of a multi-scale model, this size-range is the optimal processing unit size after a lot of derivation, and can deal with different target sizes and characteristics. In this paper, we define k as 11, that is, the filter size is 11 × 11 pixels.

The parameter θ relates to the reduction factor for image scaling. For easy understanding, the parameter θ can be regarded as the enlargement factor for the sliding 11 × 11 window. In the multi-scale model stage, there are two important parameters: the image size img × img; and the threshold value θ. Since the size of the sliding window is 11 × 11, the parameters should be set to fully cover the target when using the maximum reduction factor, where the maximum reduction factor is the result of the multiplication of $\theta_{1}$, $\theta_{2}$ and $\theta_{3}$. Through a large amount of training and derivation, we find that when the scale is too small it will result in incomplete coverage, and if the scale is too large it will result in the loss of target features. When the maximum reduction factor is close to 35, better results can be obtained in the data set. Further tests of the combination of factors in each layer achieve combinations of values of 2, 1.25 and 1.25 for the maximum reduction factor. Under this set of parameters, our algorithm can achieve a better detection effect. Of course, this specific combination is not unique, we only need an approximate optimal combination, and the parameters $\theta_{1}$, $\theta_{2}$ and $\theta_{3}$ can be exchanged freely. Therefore, in order to meet the test requirements, if we make $\theta_{1}=2,\theta_{2}=1.25,\theta_{3}=1.25$, we design a three-layer scale scaling model that can meet the full coverage requirements of all ships of different sizes, and the maximum coverage size can reach 34 pixels, which meets the test requirements of ship samples in the dataset. At the same time, because the multi-scale model uses a standard image zooming process for large object detection without any processing of the standard image for testing, it can also provide security for small-target detection (e.g., pixels covering far less than 11 × 11 of a small target in a standard image can be detected even if the target is not detected after scaling through the entire range of scales in the model), and avoid the occurrence of missed detection problems.

Since the sizes of images obtained in the detection stage are different, to facilitate subsequent processing, the first stage of this paper is to carry out image scaling. For vector images, image scaling does not cause distortion, blur or other problems, but the infrared remote sensing image is similar to a bitmap, and thus, it is necessary to select an appropriate image scaling algorithm. Bilinear interpolation is the interpolation of image pixels. Even under the condition that the original image is not smooth, bilinear interpolation will produce a smooth output, and as the infrared remote sensing image resolution is low, the target ships in the images require contour smoothing if they are small. In this case, the bilinear interpolation algorithm has a high-quality effect; the algorithm complexity is lower, and the time efficiency is better.

For point P=(x,y) on the line between $Q_{1}=\left(x_{0},y_{0}\right)$ and $Q_{2}=\left(x_{1},y_{1}\right)$, the calculation process of the y coordinate is shown in Equation (2):

(2) $$y=y_{0}+\left(x-x_{0}\right)\frac{y_{1}-y_{0}}{x_{1}-x_{0}}=\frac{y_{0}\left(x_{1}-x\right)+y_{1}\left(x-x_{0}\right)}{x_{1}-x_{0}},$$

From the perspective of the weighted average, the weight is inversely proportional to the distance between the known point and the unknown point, that is, the closer the known point is to the unknown point, the greater the weight of the solution result of the unknown point. Therefore, according to the distance between the normalised unknown point and the two known points along the *X*-axis, the two weights should be $\frac{x-x_{0}}{x_{1}-x_{0}}$ and $\frac{x_{1}-x}{x_{1}-x_{0}}$, respectively. We obtain the derivative calculation progress of y that is shown in Equation (3) as follows:

(3) $$y=y_{0}\left(1-\frac{x-x_{0}}{x_{1}-x_{0}}\right)+y_{1}\left(1-\frac{x_{1}-x}{x_{1}-x_{0}}\right)=y_{0}\left(1-\frac{x-x_{0}}{x_{1}-x_{0}}\right)+y_{1}\left(\frac{x-x_{0}}{x_{1}-x_{0}}\right),$$

Bilinear interpolation is the extension of linear interpolation in the plane region, and its core idea is to perform linear interpolation in each direction of two dimensions. Given the coordinates of four points $Q_{11}=\left(x_{1},y_{1}\right)$, $Q_{21}=\left(x_{2},y_{1}\right)$, $Q_{12}=\left(x_{1},y_{2}\right)$, and $Q_{22}=\left(x_{2},y_{2}\right),$ and given the value of function f at four points, the bilinear interpolation algorithm can be utilised to obtain the value of function f at point *P*(*x*, *y*). The calculation process is shown in Equations (4)–(6):

(4) $$f\left(x,y_{1}\right)=\frac{x_{2}-x}{x_{2}-x_{1}}f\left(Q_{11}\right)+\frac{x-x_{1}}{x_{2}-x_{1}}f\left(Q_{21}\right),\;$$

(5) $$f\left(x,y_{2}\right)=\frac{x_{2}-x}{x_{2}-x_{1}}f\left(Q_{12}\right)+\frac{x-x_{1}}{x_{2}-x_{1}}f\left(Q_{22}\right),\;$$

(6) $$f\left(x,y\right)=\frac{y_{2}-y}{y_{2}-y_{1}}f\left(x,y_{1}\right)+\frac{y-y_{1}}{y_{2}-y_{1}}f\left(x,y_{2}\right),\;$$

In this paper, a bilinear interpolation algorithm is used to expand and shrink the image size of the module to be detected, and images of different sizes are obtained as the input of the subsequent detection module. Through this scaling process, the subsequent detection algorithm has better adaptability to target ships with different characteristics. A summary of the multiscale model’s operation is shown in Algorithm 1.
**Algorithm 1** Multiscale model operation**Input:** Blocks of images, location to be processed, down-sampling parameter θ **Output:** “ResamImg” represents the output layers after down-sampling 1. **Do** the following steps: 2. Initialise parameters of first layer θ = 2, initialisation Loc.x, Loc.y, Loc.height, Loc.width, and ResamImgylen; 3. Update ResamImg as following condition: for lav = 1  pos = detector (ResamImg, model, Opts);  Loc.x = [Loc.x, pos.x × scale + xstart];  Loc.y = [Loc.y, pos. y × scale + ystart];  Loc.height = [Loc.height, pos. height × scale];  Loc.width = [Loc.width, pos. width × scale];  ResamImg_xlen = round(ResamImg_xlen/Opts.sStep);  ResamImg_ylen = round(ResamImg_ylen/Opts.sStep);  ResamImg = imresize (ResamImg, [ResamImgylen, ResamImg xlen],’bilinear’);  scale = 2;   end 4. **End**

#### 3.1.2. Regional Gradient-Feature Reconstruction

The target in the image usually has a well-defined contour, while most backgrounds do not. According to the difference between the target and the background, the background and target can be distinguished by gradient features.

To quantify the possibility of targets being contained in a region, the region size is adjusted to a fixed size, and then the gradient of the whole region is calculated as the feature vector. For image I, I (i, j) represents the grey value at position (i, j) in the image. The gradient $x_{i}$ of image I along the x-direction is the first derivative of the image in the x-direction. The gradient $y_{i}$ of image Img along the y-direction is the first derivative of the image in the y-direction. The calculation of gradients $I_{x}$ and $I_{y}$ is shown in Equations (7) and (8), respectively:

(7) $$I_{x}=\frac{I(i+h,j)-I(h,j)}{h}$$

(8) $$I_{y}=\frac{I(i,h+j)-I(h,j)}{h}$$

In the actual algorithm design process, the value of h is generally 1, and the Sobel operator is applied to quickly extract image gradient features. Sobel operator is a discrete difference operator, which is used to approximate the gray-level from an image brightness function. Using this operator at any point in the image will produce the corresponding grayscale vector. The Sobel operator is based on the gray-weighted-difference between the upper, lower, left, and right adjacent points of a pixel. If the gradient value of a pixel in the overall x and y directions is obtained, it only needs to add the gradient calculation results calculated by Sobel in each direction. The Sobel operator is used to calculate the gradient characteristics of the image, which is shown in Equations (9) and (10) as follows:

(9) $$I_{x}{=S}_{x}⊗\mathrm{Img}$$

(10) $$I_{y}{=S}_{y}⊗\mathrm{Img}$$

where Img represents the input image, $S_{x}$ and $S_{y}$ represent the Sobel operators, and ⊗ represents the gradient operation. The gradient amplitude M (i, j) of image Img at (x, y) can be obtained from $I_{x}$ and $I_{y}$, which are shown in Equation (11):

(11) $${M(i,j)=\min(|I}_{x}{(i,j)|+|I}_{y}{(i,j)|,S}_{image})$$

**Figure 2:**
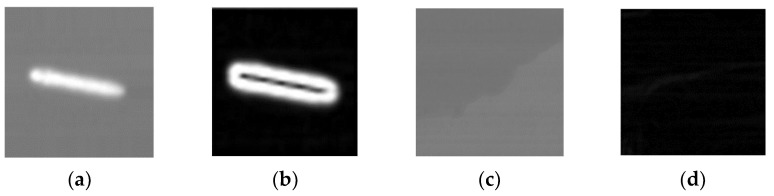
Diagram of image gradient operation: (**a**) Original image with target ship, (**b**) image with target after gradient operation, (**c**) original image with background, and (**d**) image with background after gradient operation.

Figure 2 shows a schematic of the image after scaling. The target ship can be better represented by selecting an appropriate scaling factor.

#### 3.1.3. Vector Binarization of Flow Convolution

The operation of linear convolution is essentially the computation of the inner product of two vectors by representing a vector as the weighted sum of multiple binary vectors, consisting of −1 and 1, such that the inner product operation of a vector can be computed quickly using simple bit operations. The convolution operation is an important step in the realisation of whole-target detection. When hardware is employed to realise the convolution operation, it is necessary to identify a scheme that accurately realises the convolution operation. The operation does not occupy too much space and is fast enough to complete the convolution operation of a target region in a pixel-clock. During the convolution operation, it is necessary to know the value of pixels around the current pixels. However, in the process of image processing, the data obtained exhibit the form of pixel streams rather than the whole image, and thus, it is necessary to cache the surrounding pixels that are needed. Algorithm 2 shows a summary of the vector binarisation operation.
**Algorithm 2** Vector binarisation operation**Input:** Vector w to be approximated, number of binary vectors $N_{w}$ **Output:**$\{\{\partial_{j}{\}}_{j=1}^{N_{w}}\}$, $\{\{\beta_{j}{\}}_{j=1}^{N_{w}}\}$, represents $N_{w}$ binary vectors and corresponding weights 1. **Do** the following steps: 2. Initialise residuals ε=w 3. Update $\partial_{j}$ and $\beta_{j}$ as the following conditions: for j = 1 to $N_{w}$ do   $\partial_{j}$ = sign(ε)   $\beta_{j}=<\partial_{j},\varepsilon >/||\partial_{j}{\left|\right|}^{2}$   $\varepsilon \leftarrow \varepsilon -\beta_{j}\alpha_{j}$   end 4. **End**

The approximate representation of w can be obtained through the vector binarisation approximation algorithm, $w\approx \sum_{j=1}^{N_{w}}\beta_{j}\partial_{j}$, assuming that the region size is 8 × 8. The gradient feature is a 64-dimensional feature vector, $x\in R^{64}$. The model parameters of the linear support-vector-machine (SVM) classifier based on gradient-feature training are equivalent to the gradient feature dimension, which is also a 64-dimensional vector, and is defined as $w\in R^{64}$. In the above summary of Algorithm 2, $\partial_{j}$ represents the binary basis vector $\partial_{j}\in {\{-1,1\}}^{64}$. After the binary approximation algorithm, there are a total of $N_{w}$ binary basis vectors, and $\beta_{j}$ represents the weight coefficient corresponding to the basis vector $\partial_{j}$.

**Figure 3:**
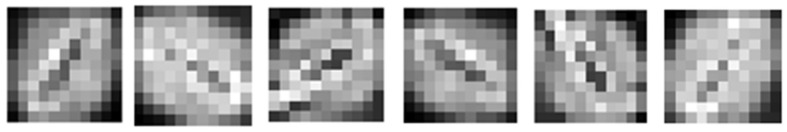
Diagram of six templates with different directions in the convolution module.

The pixels around the target pixels are stored in registers according to their addresses. During the algorithm’s operation, values flow from left to right and top to bottom throughout the image as required. The next step is to convolve the pixel value stored in the register with the corresponding convolution-kernel weight. The ping-pong operation is a commonly used data-flow control-processing technique, the main process is to assign input data streams to different data buffers isochronously through an input-data selection unit. The ping-pong operation sends the buffered data-stream to the processing module continuously for calculation by an input-data selection unit and an output-data selection unit, switching with each other according to the beat. Because the input-data- and output-data-flows are continuous, it is very suitable for the pipeline processing of data-flows to complete seamless buffering and processing of data, greatly saving buffer space. In this paper, the number of convolution kernels is constant, a new pixel can be input in a pixel-clock-cycle, and the convolution result can be output after several clock-cycle delays. If the ping-pong operation is not used, the data preprocessing module will become the bottleneck to limit the system-data-throughput in the design. By optimizing the ping-pong operation’s design, the computation period is increased. The data throughput of the system can be improved through the cache design optimisation while increasing the data buffer delay. This process can be defined as follows:

(12) $${\mathrm{Conv}=\mathrm{Max}(\mathrm{conv}(T}_{\mathrm{xn}}{,C}_{\mathrm{xn}})$$

where $T_{\mathrm{xn}}$ represents the pixel matrix, which is processed according to the regional gradient, $C_{\mathrm{xn}}$ represents the convolution template, the templates in this article are 11 × 11 pixels, and there are six template types in different directions, as shown in Figure 3. Max represents the maximum pixel matrix after convolution of the image matrix and several pixel matrices.

The value of each pixel of Conv is compared with the detection threshold ξ. A value greater than ξ is regarded as a suspected target ship, and the coordinate information of the target is output. Otherwise, it is regarded as a nontarget point. To choose an appropriate value of η, the recall rates are counted with different choices of ξ, which are shown in Table 1. We can see that a value of ξ = 0.6 is a turning point in the recall rate. When ξ is set larger than 0.6, the recall rate quickly decreases. On the other hand, when ξ is set smaller than 0.6, it will slow down the subsequent processes and bring additional false-positives. So, we choose η = 0.6 in our method as this is the optimal parameter-solution proved by a large number of tests. In addition to ensuring that the recall rate of the algorithm remains within an appropriate range, the real-time processing performance of the algorithm is maximally improved. The target information output by the threshold model is regarded as the input for region classification, in which the target information mainly includes an x-coordinate, a y-coordinate, a width and a height.

### 3.2. Multiple-Feature Fusion Classification

**Figure 4:**
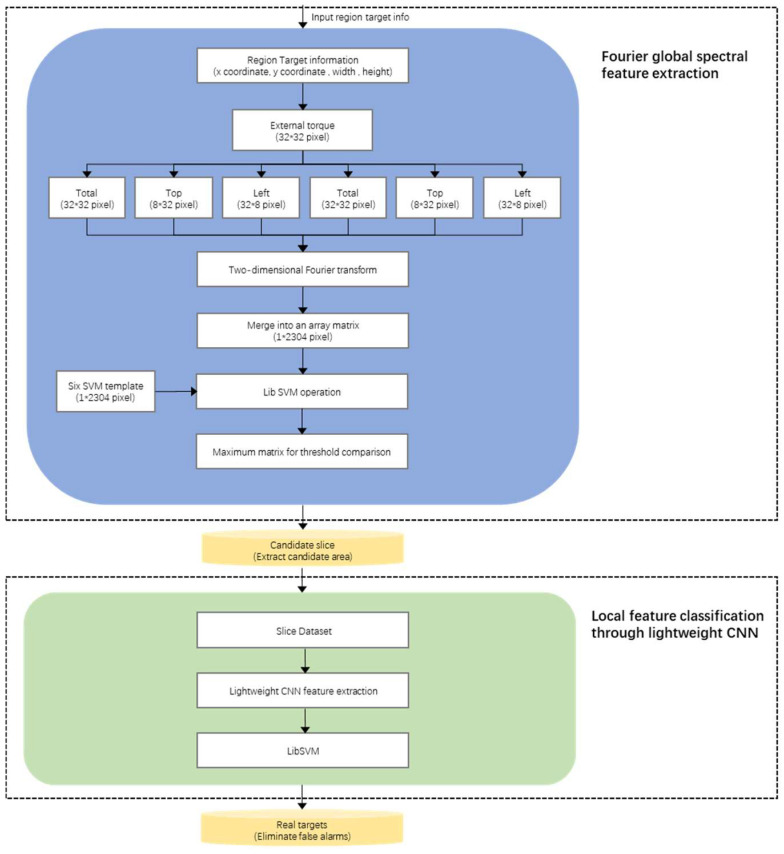
Schematic of the multiple-feature fusion classification process.

To solve the problem of complete extraction of the ship region, the suspected region of the target ship is located step-by-step based on the idea of coarse-to-fine detection, as is shown in Figure 4.

We design the multiple-feature classification process through two parts. The first is Fourier global spectral feature extraction. The global spectral features based on Fourier transform are applied to extract the Fourier features of positive and negative samples to train the classifier. The magnitude of the gradient generated by the Fourier global spectral feature represents the difference between a point in the image and its neighbourhood, which can better distinguish the target ship from the ocean background and initially exclude false-alarm. Secondly, local feature classification through lightweight CNN is undertaken. Through the full analysis of target ships in infrared remote sensing images, available features that can effectively distinguish target ships from typical false-alarm (clouds, tracks, etc.) are selected. The optimal feature-subset of the target can be constructed to quickly and accurately eliminate false-alarm to improve the accuracy and universality of ship detection.

#### 3.2.1. Fourier Global Spectral-Feature Extraction

**Figure 5:**
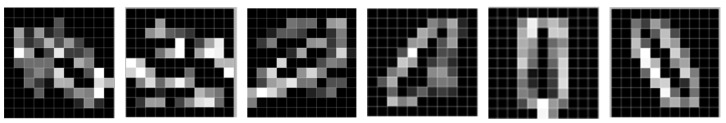
Diagram of six templates with different directions in Fourier module.

Through the function of frequency domain analysis, we can change the angle and improve the visibility of certain signal information. For example, by Fourier transform [35,36], if the numerical value is graphed and existing spikes are visible, they not only represent hidden frequencies in the mixed signal but also can solve the problem of distinguishing between several signals. Specifically, the work of this stage is to perform Fourier transform on the candidate slices obtained in the previous stage. For each candidate target image, the spatial domain is converted to the frequency domain. In other words, a two-dimensional Fourier transform is applied to each candidate region to obtain the spectral domain, which is the distribution of the image gradient. The magnitude of the gradient represents the strength of the difference between a certain point on the image and the neighbourhood point, which is used to better distinguish the target ship from the ocean background. During the training, the Fourier transform is extracted from the positive sample set of six different directions (ship direction up, down, left, right, left tilt 45 degrees, and right tilt 45 degrees) to extract the spectrum characteristics. The diagram of six templates is shown in Figure 5.

**Figure 6:**
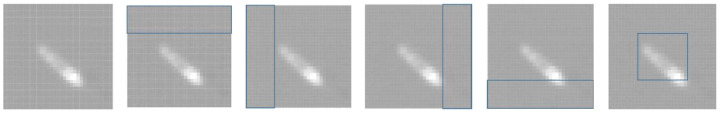
Diagram of six parts ofimage block. From left to right is the global part, the upper part, the left part, the right part, the lower part, and the middle part.

More specifically, each sample slice is extracted from six parts of theimage block, among which, the first part is the whole area of the square data block, the second part is the upper part of the square data block (width 32, height 8), the third part is the left part of the square data block (width 8, height 32), the fourth part is the lower part of the square data block (width: 32, height: 8), the fifth part is the right part of the square data block (width: 8, height: 32), and the sixth part is the middle part of the square data block (width: 16, height: 16). These specific extracted features are shown in Figure 6.

To be more specific, each sample section is extracted from the spectral features of one global region, four background regions, and one central region to which the target belongs. The two-dimensional Fourier transform is defined as follows:

(13) $$F(u,v)=\sum_{x=0}^{M-1}\sum_{y=0}^{N-1}f(x,y)e^{-j2\pi (\frac{ux}{M}+\frac{vy}{N})}$$

where u and v are frequency variables, the value of u ranges from 0 to M − 1, and the value of v ranges from 0 to N − 1. F(x, y) represents the graph function, and M, N represents the length of the sequence f(x, y). The feature descriptions of the entire global slice $I_{1}$, the centre region $I_{2}$ containing the target ship, and the background region $I_{3}$ except the centre are $f_{1}$, $f_{2}$, and $f_{3}$. The calculation process refers to Equation (14) as follows:

(14) $$f_{i}=A\left(\zeta \left[I_{i}\left(x\right)\right]\right),$$

Among them, the value of i ranges from 0 to 5, and A is the Fourier transform operation. The frequency-domain descriptor F is obtained by fusing the global and local features of the image, $F=\left[f_{0};f_{1};f_{2};f_{3};f_{4};f_{5}\right]$. Similarly, we perform a similar task for a negative sample set. The spectrum feature vectors obtained by the Fourier transform of the positive and negative sample sets in the abovementioned steps are sent to a classifier. In the experimental process, Fourier global features are applied to roughly eliminate false-alarm and extract several candidate areas. For easy understanding, the six-part solution of the Fourier transform is shown in Algorithm 3.
**Algorithm 3** Fourier operation**Input:** Original image (img) to be approximated.
**Output:** Final vector feature, number of binary vector $N_{f}$. 1. **Do** the following steps: 2. Initialise residual feature (i) 3. Update feature (i) as the following conditions: im = im2double(im); IM = abs(fft2(im)); feat0 = IM (:)’; feat0(1) = 0; IM = abs (fft2(im (1:10:))); feat1 = IM (:)’; feat1(1) = 0; IM = abs (fft2(im (:1:10))); feat2 = IM (:)’; feat2(1) = 0; IM = abs (fft2(im (end-7:end:))); feat3 = IM (:)’; feat3(1) = 0; IM = abs (fft2(im (:end-7:end))); feat4 = IM (:)’; feat4(1) = 0; IM = abs (fft2(im (end/4+1:end/4×3, end/4+1:end/4×3))); feat5 = IM (:)’; feat5(1) = 0;  feat = [feat0, feat1, feat2, feat3, feat4, feat5];  feat = feat./(sum(feat)); 4. **End**

The global Fourier transform was applied to each candidate frame to obtain the corresponding feature description, achieve a rough classification of candidate regions, and then the local feature was extracted by the lightweight CNN model to further eliminate false-alarm in the following section.

#### 3.2.2. Local Feature Classification through Lightweight CNN

Generally, when deep learning methods are utilised to extract features for classification, deeper network layers and larger datasets are needed to achieve higher classification performance. However, the larger the model, the larger the number of parameters and computing resources that are consumed. It is difficult to meet the limited resource requirements of the onboard processor. Therefore, to balance accuracy, speed and memory, the global features of positive and negative samples are extracted with the Fourier spectrum features in the previous part to roughly eliminate false-alarm in the candidate region of the target ship. A lightweight classification network is then designed to accurately identify the local features of the ship. The reason for combining the Fourier global features with local features extracted from lightweight networks in this chapter is to consider the following two aspects. (a) In general, the full connection layer in deep networks learns global patterns from the feature space, and the convolution layer learns local patterns, while lightweight networks can only learn simple features at a lower level. (b) With an increase in the network layer number, the receptive fields are also increased gradually. If the network layer is low, the convolution kernels are lower, and the receptive fields are unable to capture the global image. Therefore, the Fourier spectrum of global features can effectively reduce the size of the previous stage to produce the number of invalid candidate areas, reducing the complexity for the lightweight classification-network classification target.

**Figure 7:**
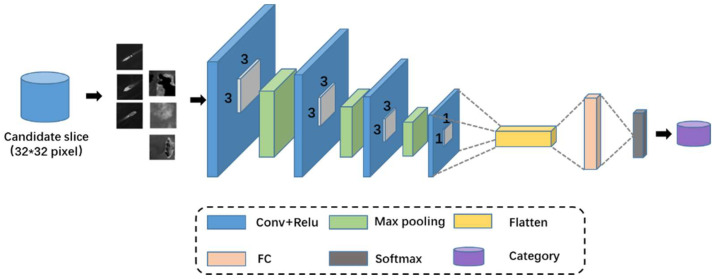
Schematic of feature extraction through a lightweight CNN.

The target ship itself in the external remote sensing image has a small scale of approximately 10 to 50 pixels. Considering the small scale of the target ship itself, the model compression will compensate for the loss of accuracy. Therefore, lightweight networks with fewer network layers are designed in this chapter. The lightweight classification network consists of four convolutional layers and two fully connected layers. The network structure is shown in Figure 7. Among them, the first three convolutional layers are configured with a convolution kernel with a size of 3 × 3, followed by the maximum pooling layer of down-sampling. The last convolution layer is configured as a 1 × 1 convolution kernel. Deep convolution and 1 × 1 convolution can reduce the computational complexity of the model with a small precision loss. These convolutional layers are deployed on rectified-linear activation-unit (ReLU) activation functions to make the network undisturbed by vanishing gradients compared to sigmoID activation functions and tanh activation functions. In addition, different data enhancement strategies are adopted in the training process. The last fully connected and softmax layers address the classification problem based on the features extracted from the previous convolutional layer. During the experiment, cross-entropy was employed to define the loss function as follows:

(15) $$\mathrm{Loss}\left(Y,P\right)=-\frac{1}{N}\overset{N-1}{\underset{i=0}{\sum }}\overset{2}{\underset{K=1}{\sum }}\left(y_{\mathrm{ik}}\log\left(p_{\mathrm{ik}}\right)\right),$$

where N is the batch size, which is 128 in this experiment. The term i represents the label of the candidate slice, $y_{ik}$ represents that the candidate slice label of block I belongs to category K, and $p_{ik}$ represents the predicted probability that the candidate slice belongs to category K. In order to facilitate understanding, the extraction and calculation of feature vectors at each layer are briefly shown in Algorithm 4.
**Algorithm 4** Local feature extraction**Input:** N training pictures of size m × n, **Output:** Parameters and thresholds for each classifier comprise the output 1. **Do** the following steps: 2. Initialise and normalize training image size and the number of filters; 3. N training pictures can be obtained: $I=[{\bar{I}}_{1},{\bar{I}}_{2},{\bar{I}}_{3}\ldots {\bar{I}}_{n}]$, where I is an image obtained after rearrangement and preprocessing of each image. 4. Filter at every stage can be expressed as: $\psi_{f}^{1}=e_{f}(\mu^{T}),f=1,2,3...L_{1}$, where $e_{f}(\mu^{T})$ denotes the f principal eigenvector of $\mu^{T}$; 5. Each obtained image was preprocessed, and the results of image segmentation were merged together, compute the block result of N pictures and one of the filter convolutions $\varphi^{i}=[\varphi^{1},\varphi^{2}\ldots \varphi^{L_{1}}]$ 6. By solving the eigenvector of $\varphi \varphi^{T}$, the feature vector corresponding to the second largest eigenvalues was taken as a filter, $\psi_{f}^{2}=e_{f}(\varphi \varphi^{T}),f=1,2,3...L_{2}$; 7. Similarly, we can calculate: $\psi_{f}^{3}=e_{f}(\varphi \varphi^{T}),f=1,2,3...L_{3}$, $\psi_{f}^{4}=e_{f}(\varphi \varphi^{T}),f=1,2,3...L_{4}$ 8. Through spatial pyramid pooling, hash coding, etc., the feature vector of each training image was obtained. 9. Input the trained feature vectors into the LibSVM to train and test them 10. **End**

This section proposes a lightweight convolutional network based on the target candidate region under resource constraints in real scenes. Based on the multi-scale target candidate-region extraction model, the network orders the candidate region to identify the real target and false-alarm, returns the index corresponding to the correctly classified candidate region to the corresponding connected region in the original image, and realises the target location according to the largest connected region’s outer rectangle. In this way, the regressive operation of object detection based on deep learning is avoided, and the amount of computation is doubled. Therefore, the computational complexity of the model with a small precision loss can be reduced.

### 3.3. Classifier Training and Target Confirmation

As a powerful classification method with the ability to minimise the classification error rate and maximise generalisation, the basic working principle of the SVM is described as follows [37]: two kinds of samples that are linearly indivisible in the input space are mapped to a high-dimensional feature space by a kernel function, and linearly constrained quadratic programming is solved in the high-dimensional feature space to obtain a classification hyperplane with a maximum interval that can linearly divide the samples.

For the dataset ${T=\{(x}_{1}{,y}_{1}{),(x}_{2}{,y}_{2}),\ldots {,(x}_{m}{,y}_{m})\},\mathrm{yi}\in \left\{-1,+1\right\}$ and the hyperplane (w, b), the geometric interval of sample points ${(x}_{i}{,y}_{i})$ is defined as $\gamma_{i}=y_{i}(w/||w||\cdot x_{i}+b/||w||)$, and the minimum geometric interval of the hyperplane (w, b) with respect to the training dataset T is $\gamma =\underset{i=1,2,\ldots ,N}{\min}\gamma_{i}$. Solving a separated hyperplane with the maximum geometric interval requires the maximum and minimum geometric interval, which can be expressed as the constrained optimisation problem $\underset{w,b}{\max}\gamma$. Considering the relationship between the geometric interval and the function interval, linearly separable support vector machines can be transformed into an optimisation problem as follows:

(16) $$\underset{w,b}{\min}\frac{1}{2}||w|{|}^{2}+s.t.y_{i}(w\times x_{i}+b)-1\geq 0(i=1,2,\ldots ,N)$$

For linear indivisibility, a soft interval can be considered to allow some samples to fail to meet constraint conditions; the optimisation objective function is then defined as follows:

(17) $$\underset{w,b}{\min}\frac{1}{2}||w|{|}^{2}+C_{p}\sum_{i=1}^{n}\xi_{i},\;s.t.y_{i}(w\times x_{i}+b)\geq 1-\xi_{i}$$

where C represents the degree of punishment for the right and wrong samples, and the optimisation of the whole algorithm can still use the Lagrange multiplier method. It can be seen from the optimisation objective function that an SVM ultimately achieves a compromise between the maximum classification interval and the minimum classification error, and its punishment is the same for positive and negative classification errors. In the process of extracting potential regions of ships, the classifier must ensure a high recall rate to avoid missing detection as much as possible. Therefore, the risks brought about by classifying ships as backgrounds and classifying backgrounds as targets differ, and the misclassification of the two cannot be treated equally. According to this feature, this paper demonstrates how the optimisation objective function improves and that the constructed-risk unbalanced SVM classifier overcomes the shortcomings of the traditional SVM classifier. In addition, the risk-unbalanced SVM classifier is applied to the task of ship potential-extraction. The optimisation objective function of an SVM with uneven risk is shown as follows:

(18) $$\underset{w,b}{\min}\frac{1}{2}||w|{|}^{2}+C_{p}\sum_{y_{i}=1}\xi_{i}+C_{n}\sum_{y_{i}=-1}\xi_{i},s.t.y_{i}(w\times x_{i}+b)\geq 1-\xi_{i}$$

where $C_{p}$ and $C_{n}$ are the risk of positive samples and the risk of negative samples, respectively, and generally $C_{p}$ > $C_{n}$. The flow of the cascade classifier training algorithm is shown in Algorithm 5.
**Algorithm 5** Cascade classifier training algorithm**Input:** Training set $T_{1}{=\{(x}_{1}{,y}_{1}{),(x}_{2}{,y}_{2}),\ldots {,(x}_{m1}{,y}_{m1})\}$, where $x_{i}\in X,y_{i}\in Y=\{-1,+1\}$, given the weights of the two classes $C_{p}$,$C_{n}$ and the minimum recall rate $d_{\min}$ **Output:** Parameters and thresholds for each classifier comprise the output 1. **Do** the following steps: 2. Calculate the variance of each sample in training set $T_{1}{=\{(x}_{1}{,y}_{1}{),(x}_{2}{,y}_{2}),\ldots {,(x}_{m1}{,y}_{m1})\}$ 3. Through all positive samples in the training set, adjust the threshold to make the recall rate $d_{1}$ of the classifier meet $d_{1}\geq d_{\min}$ 4. Eliminate the training samples marked as background by variance classifier from the negative sample set of the training set $T_{1}{=\{(x}_{1}{,y}_{1}{),(x}_{2}{,y}_{2}),\ldots {,(x}_{m1}{,y}_{m1})\}$. The training set becomes $T_{2}{=\{(x}_{1}{,y}_{1}{),(x}_{2}{,y}_{2}),\ldots {,(x}_{m2}{,y}_{m2})\}$ 5. Calculate the gradient characteristics of each sample in the training set $T_{2}{=\{(x}_{1}{,y}_{1}{),(x}_{2}{,y}_{2}),\ldots {,(x}_{m2}{,y}_{m2})\}$ 6. Train a linear SVM classifier according to the gradient characteristics of positive and negative samples of the training set $T_{2}{=\{(x}_{1}{,y}_{1}{),(x}_{2}{,y}_{2}),\ldots {,(x}_{m2}{,y}_{m2})\}$ and $C_{p}$, $C_{n}$. Adjust the threshold to make the recall rate $d_{2}$ of the classifier meet the requirement $d_{2}\geq d_{\min}$ 7. Output the parameters and classification threshold of the cascade classifier 8. **End**

For the same target, the classification algorithm may identify several bounding boxes. One target corresponds to multiple bounding boxes, meaning that it is necessary to filter out the redundant windows and retain only the optimal bounding boxes. Non-maximum suppression searches for the local maximum value of all bounding boxes to identify the maximum value within a certain neighbourhood retains the windows with the highest score in the neighbourhood and inhibits windows with low scores to filter part of the bounding boxes to improve the final detection accuracy. 

Non-maximum suppression is an iteration-ergo-elimination process; the specific algorithm is expressed in Algorithm 6.
**Algorithm 6** Improved NMS algorithm**Input:** Collection of bounding boxes $B=\{b_{1},b_{2},\ldots ,b_{n}\}$, the score of the bounding box $S=\{s_{1},s_{2},\ldots ,s_{n}\}$, and the threshold value $N_{t}$ **Output:** D, S 1. **Do** the following steps: 2. Initialize D={} 3. While B is not empty compute the index of the maximum value in S: m←argmaxS  Compute the corresponding bounding box: $M\leftarrow b_{m}$ 4. Update s, D, B and S as the following condition: D←D+M,B←B−M for $b_{i}$ in B, do if iou($b_{i}$,M) is present,  update $S\leftarrow S+s_{i},B\leftarrow B-b_{i}$ end 5. **End**

The traditional non-maximum suppression (NMS) algorithm generates a series of detection candidate boxes B and the corresponding probability value S. First, we sort the probability values and select the candidate box M with the maximum probability before M joins the final detection result set D and deletes it from B. Candidate boxes in set B whose overlap with candidate box M is greater than the threshold Nt are deleted. The main problem of the algorithm is that the adjacent detection frames must be deleted. In this case, if ships are clustered and distributed, there is an overlap between two detection frames, which causes the detection failure of ships near each other and reduces the detection accuracy of the algorithm.

To solve this problem in NMS, this paper adopts a probabilistic reset strategy to improve NMS [38]. In this algorithm, the probability of an overlapping detection frame is directly obtained by an attenuation function rather than zero. If a detection frame overlaps greatly with M, it has a very low probability of still existing. In contrast, if the detection frame only overlaps to a small extent with M, its detection probability is not affected. Its probability reset function is shown as follows:

(19) $$S_{i}=\left\{ \begin{array}{ll} S_{i} & iou(M,b_{i})<N_{t}\\ S_{i}(1-iou(M,b_{i})) & iou(M,b_{i})\geq N_{t} \end{array}\right.$$

## 4. Performance Evaluation

### 4.1. Experimental Setup

#### 4.1.1. Dataset Description

Data are the core of artificial intelligence research, and labelling data is sometimes more important than algorithms. Existing remote sensing image datasets are private datasets. Because of sensitive data or copyright issues, many private ship datasets are difficult to disclose. To promote ship detection research, this paper proposes and establishes a set of standard, infrared remote sensing image target-ship datasets. The data are mainly derived from the image data collected by a satellite, which has an important experimental reference value. Ships in the dataset have been marked in the form of rectangular frames. In different scenarios, target ships have different sizes, directions and interferences. To verify the anti-interference ability of the algorithm, the dataset should include samples of various scenarios, such as cloud interference samples, trail interference samples, reef interference samples, and sea-clutter interference samples to make the dataset persuasive.

The images employed in the experiment include ocean scenes and nearshore scenes, which have not only calm and undisturbed sea state backgrounds but also complex backgrounds, such as clouds and reefs. The ship length is various and the ship azimuth angle is arbitrary, which is suitable for the comprehensive testing and comparison of the algorithm performances. There are 214 images in the test set, including 1270 ships. This paper enlarges the dataset by following methods: Random clipping, where a fixed size image block is intercepted randomly from the original image, and the ships in the image block have been marked in the form of a rectangular box; Mirror flip, through horizontal flip and vertical flip to construct a new dataset; Rotation transformation, where the whole image rotates in a specific direction around the fixed point; Enhanced contrast, where by changing the image’s gray value to improve the visual effect of the image. We divide the image into 8456 sub-images and convert these sub-images into five datasets: dataset 1 (noninterference), which includes 4129 sub-images, including 308 target ships; dataset 2 (cloud interference), which includes 2476 sub-images, including 826 target ships; dataset 3 (trial interference), which includes 1851 sub-images, including 421 target ships; dataset 4 (reef interference), which contains 1168 sub-images, including 85 target ships; and dataset 5 (cloud interference), which containes 2788 sub-images, including 428 target ships. These datasets are classified according to interference types, and interference occurs simultaneously in multiple scenarios. However, the total number of target ships is still 1270 when repeated target ships in each sub-dataset (after classification) are excluded.

In order to evaluate the stability of the dataset and its compatibility with small sample detection, we tested and verified it through cross-validation. The specific process of the cross-validation experiment is as follows: the sample is divided into 10 parts. First, K parts are randomly selected from the sample for training, and the remaining parts are tested. To ensure the accuracy of this experiment, it was repeated three times, and then the average value of the three results was taken as the accuracy of this verification. Finally, the test accuracy of different proportions of training samples from the entire sample set is obtained, which can be used to evaluate the accuracy of the algorithm more accurately. The accuracy of the training samples is stable, which proves that the proposed method has good target expression characteristics and can effectively distinguish the target and background. At the same time, when the size of the training set exceeds 50% of the total number of samples, the test accuracy is basically stable at over 98%. 

#### 4.1.2. Applicable Platform

As the geometric progression of remote sensing image data increases and the complexity of intelligent processing algorithms increases, it is more and more difficult to process remote sensing images in real-time on the satellite platform with strictly limited resources. For large-scale remote sensing images, spaceborne resources have high requirements on algorithm running time, storage space resources and detection performance. At present, the cooperative realization of a space-borne infrared ship detection system based on DSP and FPGA has become the mainstream, but it faces two challenges: (a) it is limited by the complex space environment and hardware platform in terms of volume, weight, power consumption, transmission bandwidth alongside other aspects; and (b) satellite processors are slow to update due to the reliability, stability and cost of the equipment, which will result in their performance generally being lower than mainstream processors.

The test and verification platform of the target-detection algorithm proposed in this paper is a Tesla k40 M GPU equipped with 64 GB memory, an Ubuntu16.04 operating system, and the MATLAB 2016 language. This test environment is mainly used to help us train the models and verify the performance of modules. As a large number of templates need to be trained in the algorithm, this test environment is needed in order to ensure the accuracy of the training results. According to the actual demand for infrared target detection, this algorithm will eventually be carried out on the FPGA platform. For example, when we select the xc7k410t from Xilinx as the core processing module of the image processing unit, it is difficult to meet the deployment requirements of deep learining methods, mainly because the compression method of deep neural networks has higher resource requirements, especially for large networks. Moreover, it is difficult to design a state-of-the-art machine for data-flow scheduling for different layers, and there will be considerable redundancy in logical resources. Therefore, deep learning methods achieve good detection accuracy in natural image target detection, but they also have great limitations during image processing with limited resources. This is the advantage of the lighter network model designed in this paper under the condition of ensuring the detection rate.

The storage space of the network model designed in this paper is less than 25 MB, which meets the requirement of limited space-borne resources and provides a feasible scheme for real-time satellite image processing. Considering the low power consumption of FPGAs, the algorithm proposed in this paper provides a feasible solution for deploying deep learning networks on satellite-borne FPGAs with guaranteed accuracy.

### 4.2. Effectiveness of Our Method

#### 4.2.1. Detection Performance Verification

In this paper, the main indicators employed in the algorithm performance verification include algorithm recall rate R, algorithm accuracy P and algorithm error rate E, in which the algorithm error rate is the sum of the error rate L and error rate F, which can effectively reflect the robustness of the algorithm. The calculation process of each indicator refers to Equations (20)–(24) as follows:

(20) $$R=\frac{D_{c}}{T_{s}},$$

(21) $$P=\frac{D_{c}}{D_{s}},$$

(22) $$F=\frac{D_{f}}{T_{s}},$$

(23) $$L=\frac{D_{l}}{D_{s}},\;$$

(24) E=F+L,

where $D_{c}$ represents the number of correctly detected target ships and $D_{f}$ represents the number of falsely detected target ships. The term $D_{l}$ represents the number of missed detected target ships, $D_{s}$ represents the detected targets by different methods, $T_{s}$ represents the total real targets contained in the dataset, and the value of $T_{s}$ is 1270. This paper compares qualitative and quantitative methods with several other representative target detection methods in the dataset. The algorithm performance was verified by datasets under different interference scenarios, as shown in Table 2.

The test results show that the proposed algorithm can achieve better detection performance in the non-interference scenario, with a recall rate of 98.2%, an accuracy of 96.8% and an error rate of only 5%. It is not difficult to determine that island interference has a great impact on the algorithm in several test scenarios. The most notable reason is that the similarity among islands and ships is high, and it is difficult to distinguish between them. The test results show that the recall rate is 92.9%, that the accuracy rate is 90.9%, and that the error rate is 16.2%, which can still reach the expected performance, reflecting that the algorithm has a strong anti-interference ability.

After multi-scene verification of our algorithm, we compare five target-detection algorithms, mainly including SVDNet [39], Faster R-CNN [40], SPP-PCANet [41], RB [42], MRA [43], and DF [44]. Among them, SVDNet is designed based on the recent popular convolutional neural networks and the singular value decompensation algorithm, it provides a simple but efficient way to adaptively learn features from remote sensing images; Faster R-CNN proposes a Region Proposal Network(RPN) that shares full-image convolutional features with the detection network, thus enabling nearly cost-free region proposals. SPP-PCANet proposes coarse-to-fine ship detection strategies based on anomaly detection and spatial pyramid pooling; RB proposes a nearly closed-form ship-rotated bounding box space used for ship detection, and designs a method to generate a small number of high-potential candidates based on this space; MRA proposes a method to densely divide a test infrared image into a set of image patches and the radiation anomaly of each patch is estimated by a Gaussian Mixture Model, thereby target candidates are obtained from anomaly image patches, then target candidates are further checked by a more discriminative criterion to obtain the final detection result; DF consists of a simple region proposal network and a deep forest ensemble, among which the region proposal network, that is trained over gradient features robustly generates a small number of candidates that precisely cover target ships in various backgrounds, and the deep forest ensemble adaptively learns features from remote sensing data and discriminates real ships from region proposals efficiently.

Through the comparative analysis of the compared methods regarding their recall, precision, running time, etc. We summarize the experimental results from three parts. Compared with traditional detection methods such as RB and MRA, our method has a greatly improved detection performance and the processing unit is smoother and more efficient; compared with deep learning methods such as SVD, Faster R-CNN, SPP-PCANET and DF, our method is lighter on the premise of ensuring the detection accuracy, especially compared to the lightweight networks SVD and SP-PCANET, and shows substantial results in in terms of processing speed. In addition, there are also some space-oriented methods in the above algorithms such as SVD and MRA, despite this our method is better in terms of detection performance and processing speed, although MRA algorithms may have fewer resources because of their lack of networks. The performance comparison results are shown in Table 3. In brief, our approach is more effective at ship detection than the other three methods and takes less time to process an image.

**Figure 8:**
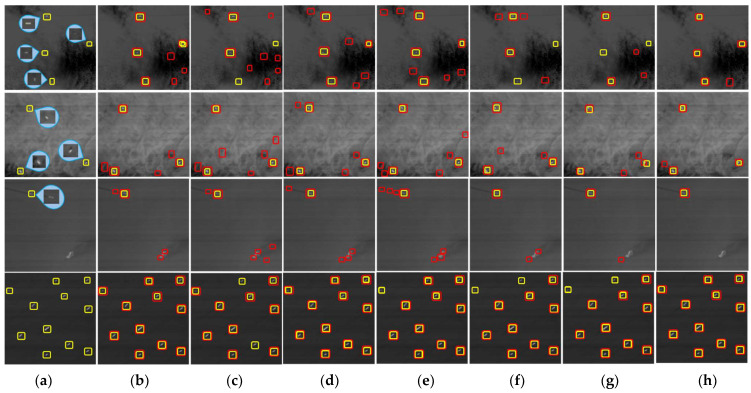
Detection results for various methods. (**a**) Real target in the original image, (**b**) SVD, (**c**) Faster R-CNN, (**d**) SPP-PCANet, (**e**) RB, (**f**) MRA, (**g**) DF, and (**h**) our method.

The detection results of different methods in different scenarios are shown in Figure 8. The first line indicates thin cloud interference and has four real target ships, the second line indicates the cloud interference and sea-clutter interference scenes and has three real target ships, the third line indicates the reef interference and trail interference scenes and has one real target ship, and the fourth line indicates a ship-intensive scene with eleven real target ships. As shown in Figure 8, the yellow boxes represent the real detected targets in the original image, and the red boxes represent the detected results of different methods. Even though some false-alarm are generated due to all kinds of interference, our method achieves impressive detection performance on different sea surfaces and fewer misses and errors than other algorithms. It is proven that the proposed algorithm has high stability in different scenarios.

#### 4.2.2. Robustness Verification

This algorithm is a strong, robust and effective algorithm. Specifically, robustness is mainly reflected in the following three points. Firstly, the model has high accuracy or effectiveness. Secondly, small deviations from model assumptions can only have small impacts on the algorithm’s performance. Thirdly, large deviations from model assumptions should not have a “catastrophic” impact on algorithm performance. So we verify function 1 with the F-measure, we verify function 2 with the True–False-positives rate, and we verify function 3 with Mean error rate.

- F-measure score

The precision and recall rates are sometimes contradictory, so they need to be considered comprehensively. The F-measure is the weighted average of the precision rate and recall rate, and the F-measure value is the arithmetic mean divided by the geometric mean. When the F-measure value is small, true positives increase and false-positives decrease. Therefore, we can verify the performance of the algorithm through the F-measure score that corresponds to the first of the three function-points of robustness verification above. The calculation method for the F-measure is as follows:

(25) $$F_{score}=\frac{(\alpha^{2}+1)P*R}{\alpha^{2}(P+R)}$$

where P represents algorithm accuracy, R represents algorithm recall rate, and α represents the calculation parameter and is usually set to 1.

The F-measure weighs both precision and recall, F-score comparison results of the different algorithms are shown in Table 4, where $F_{\mathrm{none}}$ represents an F-score without any interference, $F_{\mathrm{cloud}}$ represents an F-score under cloud interference, $F_{\mathrm{trail}}$ represents an F-score under trail interference, $F_{\mathrm{reef}}$ represents an F-score under reef interference, $F_{\mathrm{clutter}}$ represents an F-score under clutter interference, and $F_{\mathrm{total}}$ represents the total F-Score of detection.

By comparing the experimental results in Algorithm 6, it is not difficult to find that our algorithm can achieve a better F-score in different scenes, especially in the scene without interference; the F-score of the proposed method reaches 0.975. Moreover, by comparing experiments in full scenarios, it can be found that our method can also achieve better results, which proves the effectiveness and stability of the algorithm in different scenes.

- True–False-positives graph

After the proposal of a region, the algorithm preliminarily obtains the potential target region, removes some background interference and negative targets, and obtains the suspected positive targets. Then, real target ships, i.e., true positive targets, are screened out through region classification, and false-alarm in the suspected positive targets, i.e., false-positive targets are eliminated. This part quantifies the performance of the algorithm under different interference scenes by using the true–false-positives graph. Because different scenes have different effects on the algorithm, the curve can intuitively reflect the adaptability of the algorithm to each scene, that is, whether there will be obvious differences in the algorithm’s performance when the interference changes. Therefore, this part of the test corresponds to the second of the three function-points of the above robustness verification. The calculation of the true positives rate is as follows:

(26) $$T_{rate}=\frac{T_{P}}{T_{P}+F_{P}}$$

where $T_{\mathrm{rate}}$ means the true positives rate, $T_{P}$ means the true positives samples, and $F_{P}$ means the false-positives samples.

**Figure 9:**
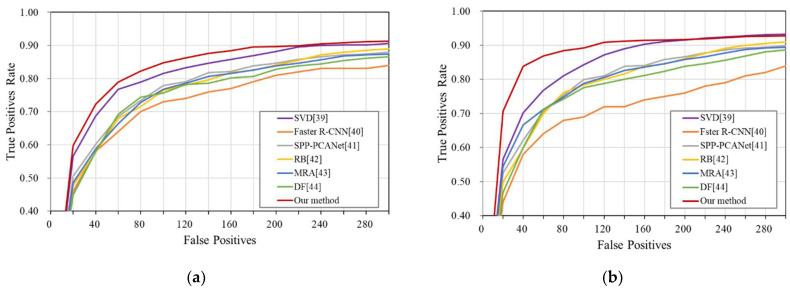

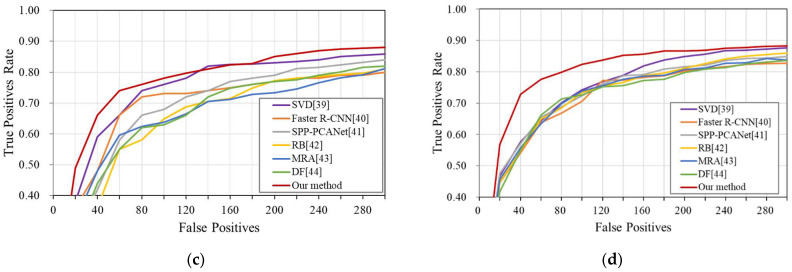
Diagram of false-positive and true-positive rates: (**a**) cloud interference; (**b**) trail interference; (**c**) reef interference; and (**d**) sea-clutter interference.

In a supplemental test, we compare the correlations between the false-positive and true positive rates of different algorithms under different interferences, as shown in Figure 9. 

These four scenarios are a cloud scenario, a trail scenario, a reef scenario and a sea-clutter scenario. Through the linear comparison of the data in the figure, our method can quickly reach the expected detection rate when generating negative samples, which indicates that the method has good robustness and anti-interference ability. The true–false-positive quantitative data under all scenarios are shown in Table 5, where $F_{P}$ means false-positives, and $T_{\mathrm{rate}}$ means the true positives rate.

- Mean error rate

The robustness of the algorithm should be compared regarding not only the detection performance but also the avoidance of false and missed detections. To better prove the robustness of the algorithm, we compare the mean error rate obtained with different image quantities, as shown in Table 6. The third function-point of robustness is to verify the impact of large deviations on the algorithm. Since the average value is considered to be a positive correlation factor of an algorithm, the mean error rate is more meaningful than any robust measure. Therefore, this part counts the average error rate in the overall operation cycle of each algorithm, and the results can reflect the stability of the algorithm during operation, The lower the mean error rate, the better the compatibility of the algorithm with strong anti-interference.

## 5. Conclusions and Future Work

This paper presents an infrared ship detection method based on the combination of traditional feature recognition and lightweight CNN classification. The effective ship candidate-region is extracted by a multiscale feature extraction model, and the global features extracted by the Fourier transform are combined with the local features extracted by a lightweight CNN to eliminate false-alarm to confirm the target ship. Compared with the existing methods, the proposed method is more efficient and robust for target detection in complex scenes. Our future work will focus on memory storage and explore hard negative mining strategies to improve the generalisation performance.

## Figures and Tables

**Table 1:** The effect of threshold on algorithm performance.

ξ	0	0.2	0.4	0.6	0.8	1
Recall rate	1	0.999	0.996	0.992	0.756	0
Time/img	1.998	1.996	1.986	1.962	1.42	0

**Table 2:** Detection results of our method under different interferences.

	None	Cloud	Trail	Reef	Clutter	Total
$T_{s}$	488	1056	658	186	524	1270
$D_{s}$	495	1095	676	189	531	1298
$D_{c}$	479	999	607	165	468	1180
$D_{f}$	9	57	51	21	56	116
$D_{l}$	16	96	69	24	63	92
R (%)	98.2	94.6	92.3	88.6	89.3	92.9
P (%)	96.8	91.2	89.8	87.2	88.1	90.9
E (%)	5	14.2	17.9	24.2	22.6	16.2

**Table 3:** Performance comparison results of the different algorithms.

	SVD [39]	Faster R-CNN [40]	SPP-PCANet [41]	RB [42]	MRA [43]	DF [44]	Ours
$T_{s}$	1270	1270	1270	1270	1270	1270	1270
$D_{s}$	1293	1483	1319	1327	1288	1277	1298
$D_{c}$	1173	1188	1153	1133	1110	1144	1180
$D_{f}$	119	253	160	185	175	132	116
$D_{l}$	98	94	121	143	162	126	92
R (%)	92.4	93.6	90.8	89.2	87.4	90.1	**92.9**
P (%)	90.7	80.1	87.4	85.4	86.2	89.6	**90.9**
E (%)	16.9	26.3	21.8	25.4	26.4	20.3	**16.2**
Time/img	3.2	3.28	3.14	5.2	4.6	2.7	**1.9**
Model (M)	28	156	32	-	-	52	**24**

**Table 4:** F-score comparison results of the different algorithms.

	SVD [39]	Faster R-CNN [40]	SPP-PCANet [41]	RB [42]	MRA [43]	DF [44]	Ours
$F_{\mathrm{none}}$	0.962	0.909	0.964	0.938	0.926	0.934	**0.975**
$F_{\mathrm{cloud}}$	0.924	0.828	0.912	0.898	0.893	0.908	**0.929**
$F_{\mathrm{trail}}$	0.901	0.826	0.884	0.846	0.862	0.886	**0.910**
$F_{\mathrm{reef}}$	0.876	0.806	0.896	0.892	0.874	0.868	**0.879**
$F_{\mathrm{clutter}}$	0.866	0.822	0.862	0.876	0.764	0.796	**0.887**
$F_{\mathrm{total}}$	0.915	0.863	0.891	0.873	0.868	0.899	**0.919**

**Table 5:** True–False-positives quantitative data under all scenarios.

	SVD [39]	Faster R-CNN [40]	SPP-PCANet [41]	RB [42]	MRA [43]	DF [44]	Ours
$T_{\mathrm{rate}}$ $(F_{P}$ = 20)	0.468	0.436	0.475	0.448	0.456	0.418	**0.568**
$T_{\mathrm{rate}}$ $(F_{P}$ = 40)	0.577	0.528	0.572	0.548	0.558	0.550	**0.728**
$T_{\mathrm{rate}}$ $(F_{P}$ = 60)	0.645	0.596	0.656	0.650	0.634	0.662	**0.776**
$T_{\mathrm{rate}}$ $(F_{P}$ = 80)	0.701	0.642	0.688	0.685	0.698	0.714	**0.799**
$T_{\mathrm{rate}}$ $(F_{P}$ = 100)	0.742	0.688	0.724	0.734	0.738	0.726	**0.824**
$T_{\mathrm{rate}}$ $(F_{P}$ = 120)	0.768	0.712	0.761	0.755	0.756	0.752	**0.838**
$T_{\mathrm{rate}}$ $(F_{P}$ = 140)	0.789	0.736	0.788	0.766	0.776	0.756	**0.852**
$T_{\mathrm{rate}}$ $(F_{P}$ = 160)	0.819	0.768	0.791	0.788	0.786	0.772	**0.856**
$T_{\mathrm{rate}}$ $(F_{P}$ = 180)	0.838	0.783	0.809	0.796	0.789	0.776	**0.866**
$T_{\mathrm{rate}}$ $(F_{P}$ = 200)	0.849	0.796	0.816	0.811	0.808	0.798	**0.867**
$T_{\mathrm{rate}}$ $(F_{P}$ = 220)	0.856	0.804	0.821	0.826	0.812	0.809	**0.869**
$T_{\mathrm{rate}}$ $(F_{P}$ = 240)	0.868	0.807	0.836	0.841	0.827	0.814	**0.875**
$T_{\mathrm{rate}}$ $(F_{P}$ = 260)	0.869	0.811	0.842	0.850	0.829	0.825	**0.878**
$T_{\mathrm{rate}}$ $(F_{P}$ = 280)	0.873	0.813	0.844	0.855	0.842	0.831	**0.882**
$T_{\mathrm{rate}}$ $(F_{P}$ = 300)	0.876	0.815	0.849	0.860	0.838	0.836	**0.883**

**Table 6:** Mean error rate comparison results of the different algorithms.

	SVD [39]	Faster R-CNN [40]	SPP-PCANet [41]	RB [42]	MRA [43]	DF [44]	Ours
Images	214	214	214	214	214	214	214
False detection	119	253	160	185	175	132	116
Missed detection	98	94	121	143	162	126	92
Error detection	217	347	281	328	337	258	208
False/image	0.56	1.18	0.74	0.76	0.82	0.62	**0.54**
Missed/image	0.46	0.44	0.57	0.67	0.75	0.59	**0.43**
Error/image	1.02	1.62	1.31	1.53	1.57	1.21	**0.97**

## Data Availability

The data presented in this study are available on request from the corresponding author. The data are not publicly available due to the sensitive issues.
